# Intra-articular hyaluronan (Synvisc®, hylan G-F 20) for the treatment of first metatarsophalangeal joint osteoarthritis: a randomised, placebo controlled trial

**DOI:** 10.1186/1757-1146-4-S1-O54

**Published:** 2011-05-20

**Authors:** Gerard V Zammit, Shannon E Munteanu, Hylton B Menz, Karl B Landorf, Christopher J Handley, Ayman ElZarka, Jason DeLuca

**Affiliations:** 1 Musculoskeletal Research Centre, Faculty of Health Sciences, La Trobe University, Bundoora 3086, Victoria, Australia; 2 Department of Podiatry, Faculty of Health Sciences, La Trobe University, Bundoora 3086, Victoria, Australia; 3 School of Human Biosciences, Faculty of Health Sciences, La Trobe University, Bundoora 3086, Victoria, Australia; 4 Southern Cross Medical Imaging, La Trobe University Private Hospital, Bundoora 3083, Victoria, Australia

## Background

The objective of this randomised controlled trial (RCT) was to comparatively assess the effectiveness of intra-articular hyaluronan (Synvisc®, hylan G-F 20) against an intra-articular sterile saline placebo for the treatment of radiographically confirmed, symptomatic first metatarsophalangeal joint (MTPJ) osteoarthritis (OA).

## Methods

One hundred and fifty one (n=151) participants with symptomatic first MTPJ OA were randomly allocated to receive up to 1ml intra-articular injection of either Synvisc®, hylan G-F 20 or a sterile saline placebo. Outcomes were evaluated at 1, 3 and 6 months post-injection. Primary outcome measurements included the *foot pain* and *foot function* sub-scales of the Foot Health Status Questionnaire (FHSQ). Secondary outcome measurements were pain at the first MTPJ during walking and rest, self-reported stiffness at the first MTPJ, magnitude of symptom change with allocated treatment, global satisfaction, health-related quality of life (assessed via the Short-Form-36, version two), first MTPJ dorsiflexion range of motion, strength of the hallux plantarflexors, use of pain-relieving medication, concomitant therapies and dynamic plantar pressures.

## Results

This RCT was completed in July 2010, with 147 (97%) participants followed up at 1 month, 127 (84%) at 3 months and 135 (89%) at 6 months. Both the placebo and treatment groups displayed improvements at the 1, 3, and 6 month follow-up periods. However, there were no statistically significant differences between the placebo or treatment groups for the primary outcome measures of FHSQ *foot pain* and *footfunction* during any review period. With few exceptions, there were no statistically significant differences in the secondary outcome measurements between the groups.

## Conclusions

A single intra-articular injection of Synvisc®, hylan G-F 20 is no more effective than a single intra-articular injection of sterile saline (placebo) in reducing symptoms and improving function in people with symptomatic, radiographically confirmed first MTPJ OA.

